# Impact of prophylaxis, inhibitors, and genetics on joint outcomes according to the IPSG-MRI score in hemophilia A, B and vWD type 3

**DOI:** 10.3389/fmed.2025.1668012

**Published:** 2026-01-27

**Authors:** Anna Seeliger, Sebastian Berg, Hannah Glonnegger, Doris Boeckelmann, Markus Uhl, Johannes Oldenburg, Axel Schlagenhauf, Barbara Zieger

**Affiliations:** 1 Department of Pediatric Hematology, Oncology and Stem Cell Transplantation, Faculty of Medicine, Children’s Hospital, Medical Center, University of Freiburg, Freiburg, Germany; 2 Division of Pediatric Radiology, Department of Radiology, University Medical Center Freiburg, University of Freiburg, Freiburg, Germany; 3 IBID – Institut für bildgebende Diagnostik, Freiburg, Germany; 4 Institute of Experimental Hematology and Transfusion Medicine, University Clinic Bonn, Bonn, Germany; 5 Division of General Pediatrics, Department of Pediatrics and Adolescent Medicine, Medical University of Graz, Graz, Austria

**Keywords:** arthropathy, hemophila A, hemophila B, inhibitors, IPSG score, joint health, prophylaxis, vWD type 3

## Abstract

**Introduction:**

Hemophilia A (HA) and hemophilia B (HB) are X-linked-bleeding disorders caused by deficiency of clotting factors VIII and IX, while von Willebrand disease (vWD) type 3 involves the lack of von Willebrand factor and FVIII. Chronic joint damage from recurrent bleeding is a serious complication.

**Aim:**

The aim was to investigate the association of prophylactical treatment, severity of the disease and joint outcome.

**Methods:**

In this retrospective, single-center study we evaluated joint health in 41 patients with HA, HB, and vWD type 3 who visited our outpatient clinic since 2000 using Magnetic resonance imaging (MRI) and applied the International Prophylaxis Study Group (IPSG) score. A total of 246 MRI images (knees, elbows, ankles) were analyzed in relation to disease severity, genetics, inhibitor-formation, and therapy.

**Results:**

Of 41 patients, 28 (68%) had severe HA or HB, 10 (24%) moderate, one (2%) mild, and two (5%) were vWD patients. 19 patients with severe HA/HB received primary prophylaxis. Inhibitors developed in 7 patients (17%), most of them had loss-of-function mutations. We observed hemophilic arthropathy in 7/39 (18%) hemophilia patients (all with severe HA/HB). Only one of the 19 patients receiving early prophylaxis developed arthropathy, in the context of inhibitor development. Minor changes (IPSG score 1–5) were observed in 20% of joints while 74% of joints showed no alterations (IPSG score 0). Only 6% of joints showed hemophilic arthropathy (IPSG score ≥ 8) with ankle joints most frequently affected (10%). Among vWD-patients, one exhibited minor changes; the other had no detectable joint damage despite vWF-inhibitor presence.

**Discussion:**

This study shows that the IPSG score is a suitable tool for assessing joint health in patients with hemophilia and vWD. Reduced joint damage was associated with early diagnosis, consistent prophylaxis, and therapy adherence.

## 1 Introduction

Characteristic of Hemophilia A (HA) and B (HB) is a reduction or total lack of the coagulation factors VIII (FVIII) and IX (FIX), respectively (1). Hemophilia is classified as being severe (<1%), moderate (1%–5%), or mild (>5%) (2). Chronic joint damage is a serious complication of hemophilia leading to significant morbidity and a lower quality of life. Chronic joint damage is mainly caused by repeated bleeding into the joints, especially ankle, knee, and elbow joints (3, 4). Patients with severe von Willebrand disease (vWD type 3) also have a severe FVIII deficiency due to the lack of vWF, leading to similar joint bleeding and arthropathy (5).

The aim of this study is to investigate the relationship between joint health assessed by the IPSG MRI scoring system and clinical parameters including disease severity, genetic mutations, inhibitor development, and prophylactic treatment in patients with hemophilia A, hemophilia B, and vWD type 3. MRI plays a central role in diagnosing and monitoring joint health.

The IPSG score is an additional assessment scale for hemophilic arthropathy that quantifies the presence of soft tissue changes (effusion/hemarthrosis, synovial hypertrophy and hemosiderin deposition) and osteochondral changes (surface erosions, erosions, cysts and cartilage degradation); it also serves to detect early joint changes. The IPSG score ranges from 0 to 17 points (0 corresponds to the joint’s best condition with no pathologies, while 17 characterizes the worst joint condition involving progressive arthropathy) (6). This scale relies on clearly defined imaging parameters; validation studies have demonstrated high inter-reader reliability, supporting its objectivity and reproducibility (7). The IPSG score is a standardized method for assessing various MRI sequences and for determining the joint health of patients with HA, HB and severe vWD. Moreover, it enables a differentiated analysis of subclinical damage.

Blood-induced joint damage is triggered by multifactorial mechanisms. Under normal conditions, synovial cells clear blood from the joint space; however, recurrent bleeding overwhelms this clearance, leading to hemosiderin accumulation that triggers synovial inflammation and proliferation, which may culminate in hemophilic arthropathy (4, 8). The main aim of any hemophilia treatment is to minimize such bleeding events and prevent the progression of joint damage (9). Comprehensive hemophilia management therefore comprises early diagnosis, educating the patient about the disease, regular prophylactic treatment, the patient’s self-managed treatment documentation, regular clinical check-ups, factor activity (trough and peak levels of FVIII and FIX) and inhibitor testing.

The treatment of HA and HB has improved in recent decades, in particular by introducing prophylactic substitution of factor VIII (for HA) or IX (for HB) mostly for patients suffering from severe hemophilia to prevent bleeding and consequently joint damage. Weight-adapted prophylactic factor therapy is administered regularly several times a week (3 times/week or 2 times/week). Over the past 15 years, it was recommended to start prophylactic treatment before the second joint bleed and as soon as mobility increased, in order to prevent joint damage. Nowadays, with the availability of emicizumab, which is a bispecific, factor VIII mimetic antibody (HAVEN 7 study) it is being discussed to start prophylaxis immediately after the diagnosis is established. Most patients with moderate or mild hemophilia are treated on demand, i.e., factor FVIII or FIX is replaced when required (in case of injury or surgery).

Several studies have demonstrated that patients with moderate and severe HA/HB undergoing prophylaxis bleed less frequently and have a better quality of life than those who are only treated on demand (5, 10). Emicizumab is administered subcutaneously and has a longer half-life, which is why it has improved both quality of life and compliance (11). The development of inhibitors to factor replacement therapy remains challenging, however, it can be treated with high-dose FVIII, bypass products (immune tolerance therapy, ITT) and for HA patients with inhibitors also with emicizumab.

Some patients still experience breakthrough bleedings despite individualized prophylactic therapies. Such bleeding is sometimes clinically obvious, but can also present as subclinical, unrecognized bleeding episode. Recurrent bleedings can exacerbate joint damage; which should therefore be diagnosed early via MRI or ultrasound techniques (11). MRI can help to detect these unnoticed bleeding episodes early so that joint damage can be identified before it progresses and causes hemophilic arthropathy.

Radiologic imaging plays a key role alongside patient-reported bleeding events (treatment documentation) and clinical examination. MRIs, ultrasound and X-ray examinations are used to detect joint changes. Nowadays, MRI and ultrasound analyses are particularly useful for identifying early, subclinical joint damage. MRI remains the gold standard for the non-invasive detection and quantification of cartilage damage, especially subchondral cysts (6). In hemophilic arthropathy, specific MRI sequences can reveal even minimal hemosiderin deposits, as well as synovial hypertrophy, cartilage defects, and bone damage. This study was performed to investigate the association of prophylactical treatment, severity of the disease and joint outcome in these patients.

## 2 Materials and methods

### 2.1 Patients

This study involving human participants was reviewed and approved by the Ethics Committee of the Albert Ludwig University of Freiburg (222/20_230103). Included were 41 patients (HA *n* = 30, HB *n* = 9, vWD type 3 *n* = 2) presenting in our outpatient clinic between 2000 and 2023 who had agreed to participate in the study and for whom MRI images were available. All 41 patients or their legal guardians gave their written consent to participate in this study. Excluded were patients who denied MRI investigations and/or did not agree to participate in the study. Biochemical and genetic data, inhibitor development and therapy regimens (including substitution calendars) were acquired from all study patients’ medical records.

### 2.2 Methods

#### 2.2.1 IPSG scoring

A total of 246 MRI scans of the knee, elbow, and ankle joints were carried out between 2000 and 2023. The MRIs had previously been evaluated by an experienced pediatric radiologist who was especially trained for joint MRI assessment. The second radiologist who retrospectively assigned the IPSG score was blinded to the original reports, thereby enabling an independent assessment. The scoring was compared with the written reports at the time of MRI investigation.

The MRI examinations were conducted on a 1.5 Tesla scanner using surface coils. Slice thickness was 3 mm, and all joints were imaged in three planes. Routine protocols included turbo spin-echo sequences with T1-weighting and fat-suppressed T2-weighting. Most patients also underwent bleeding-sensitive gradient echo sequences. In a small proportion of the scans, hemosiderin-sensitive sequences were not performed. The IPSG score does not require that bleeding be visualized using these specific sequences.

When assigning the IPSG score, the MRI images were analyzed for soft tissue changes and various joint structures including the cartilage, articular surfaces, synovial membrane, capsule and bone marrow (7, 12).

Soft tissue changes assessed within IPSG score include effusion/hemarthrosis, synovial hypertrophy and hemosiderin deposition. These pathologies are attributable to the inflammatory reaction and repeated bleeding in the joint. Scoring is from 0 to 3 points for each subcategory (Table 1). The sum of the points from the three subcategories yields an overall assessment of the soft tissue changes. A higher score indicates advanced soft tissue damage often associated with chronic inflammatory processes.

**TABLE 1:** International Prophylaxis Study Group (IPSG) scoring adapted to (7).

Soft tissue changes	subcategory	IPSG score points
Effusion or hemarthrosis	Small effusion (only visible to a limited extent)	1
	Moderate effusion (affecting several joint areas)	2
	Extensive/pronounced effusion (affecting the entire joint and potentially leading to significant joint enlargement)	3
Synovial hypertrophy	Small, punctiform thickening	1
	Moderate hypertrophy (visible in several joint areas)	2
	Large, considerable thickening (potentially impairing joint function and indicating chronic inflammation)	3
Hemosiderin deposits	Small hemosiderin deposits (visible as small areas)	1
	Moderate deposits (affecting larger regions)	2
	Large hemosiderin deposits (indicating recurrent bleeding and persistent inflammation)	3
**Soft tissue changes**	**subcategory**	**IPSG score points**
Surface erosions	Any surface erosion	1
	Severe erosions affecting half or more of the articular surface in at least one bone	2
Subchondral cysts	At least one subchondral cyst	1
	Subchondral cysts in at least two bones, or cyst changes involving a third or more of the articular surface in at least one bone (signs of progressive bone resorption)	2
Cartilage degradation	Any loss of joint cartilage height	1
	Loss of half or more of the joint cartilage volume in at least one bone	2
	Loss of the entire thickness of joint cartilage in at least some area in at least one bone (which can lead to considerably impaired function)	3
	Full-thickness loss of cartilage including at least one half of the joint surface in at least one bone	4

Osteochondral changes affecting the bone and cartilage are a significant indication of progressing hemophilic arthropathy. Grading is differentiated regarding erosions, cysts and cartilage changes and degradation. The most serious anomalies detectable during osteochondral assessment are cartilage damage or cartilage degradation (Table 1). Adding up the points from the four subcategories yields the final score reflecting osteochondral pathologies. Here, a high score indicates significant structural damage indicative of hemophilic arthropathy

The final IPSG score is the total from adding the scores for the two main categories (soft tissue and osteochondral changes). No specific hemosiderin sequence was performed in 8 MRI analyses of our cohort’s ankle joints and 9 MRI exams of the elbow joints due to external MRI images and older images in which this sequence was not standard.

### 2.3 Re-evaluation of genetic reports

We had access to original genetic reports from the years 1999 to 2023 for 38 patients; the genetic mutation was taken from medical reports for two patients. We did not have genetic analysis for one patient because he had been treated at another hemophilia center before, and the pathogenic variant has been known in his family. Based on the original genetic data re-evaluation using MANE Select Transcripts NM_000132(F8), NM_000133(F9), and NM_000552(VWF) allows assignment of causative mutations for 39 patients. We investigated the occurrence of the genetic alterations in population databases (dbSNP v156, gnomAD v2.1.1), in Clinvar and the coagulation factor variant databases of the European Association for Hemophilia and Allied Disorders (EAHAD) and classified the variants according to the guidelines of the American College of Medical Genetics (ACMG).

### 2.4 Statistical analysis

Statistical analysis was performed for the cohort of hemophilic patients (*n* = 39) excluding the two patients with vWD type 3. Descriptive statistics were calculated for all variables and reported as frequencies and percentages for categorical variables, and as median with range for the observation period. The observation period for each patient was calculated as the time difference in days between the first and last MRI examination date. To investigate associations between clinical and genetic factors and the presence of hemophilic arthropathy (IPSG score ≥ 8), Fisher’s exact tests were performed for the following categorical variables: disease severity (severe vs. moderate/mild), diagnosis type (hemophilia A vs. B), prophylaxis status (regular prophylaxis vs. other regimens), and inhibitor presence (yes vs. no). Due to the small number of patients with IPSG score ≥ 8 (*n* = 7) and the distribution across multiple mutation types, formal statistical testing for mutation type associations was not performed.

In addition, maximum IPSG component scores per patient and composite scores (sum of soft tissue and bone-joint components) were calculated to summarize the extent of arthropathy. The proportion of measurements per patient with scores greater than zero was also computed as an indicator of disease burden over time. Comparisons of these continuous variables between groups (e.g., prophylaxis status, disease severity) were performed using the non-parametric Mann-Whitney U test due to non-normal data distributions. For comparisons across multiple mutation groups, the Kruskal-Wallis test was applied to the maximum IPSG scores to assess differences in arthropathy severity by mutation type.

All statistical tests were two-sided, and a *p*-value < 0.05 was considered statistically significant. Statistical analyses were performed using Python (version 3.11) with the scipy.stats and pandas libraries.

## 3 Results

### 3.1 Patient cohort

Most of the patients in this study suffered from HA (30/41patients, 73%). Within this group, 23/41 (56%) patients with severe HA (sevHA) represented the majority, followed by 6/41 (15%) patients with moderate HA (modHA) and 1/41 (2%) patient with mild HA (mildHA). Hemophilia B (HB) was represented by a total of 9/41 (22%) patients, with 5/41(12%) patients suffering from severe HB (sevHB) and 4/41 (10%) patients from moderate HB (modHB). A total of 2/41 (5%) patients with von Willebrand disease (vWD) type 3 were also included in this study.

At the time of retrospective data analysis, the patients were between 2 and 39 years old with a median age of 20.5 years. The age range was 11 months to 21 years at the most recent MRI exam of our patients. Patients weight was assessed using age- and sex-specific percentiles: 36/41 patients were within the 3rd to 97th percentile range.

A total of 18/28 (64.3%) patients with sevHA/HB received primary prophylaxis. A total of 8/23 (34.5%) sevHA and 2/5 (40%) sevHB patients underwent no primary prophylaxis because of older age and/or of the refugee situation at their first visit in our clinic.

Six patients with modHA, one patient with mildHA and 4 patients with modHB were treated on demand. One of 6 patients with modHA was switched to regular prophylaxis during the treatment due to recurrent joint bleedings during athletic activities and trauma. In HA, prophylactic factor substitution was usually carried out with standard half-life (SHL) factor VIII 20–40 IE/kg three times a week, while in HB the same dosage of SHL factor IX was administered twice a week. About 3 years ago, many patients with HA have been switched to Emicizumab as prophylactic treatment. Non-compliance was defined as the absence of regular prophylactic treatment by the patient, lack of maintained substitution records, failure to attend scheduled follow-up visits, or non-adherence to the recommended therapy despite medical advice. Patients’s characteristics are summarized in Table 2.

**TABLE 2:** Patients’ characteristics.

Patient ID	Age at the latest MRI in yrs.	Inhibitors	Primary prophylaxis	Age at start of therapy	Medication	Switching to	Comments
sevHA1	11	No	Yes	1 mo.	psFVIII SHL	Emicizumab	Low dose therapy first yr.
sevHA2	15	Yes, low	Yes	1 yr.	rFVIII SHL		
sevHA3	11	No	Yes	3 yrs.	psFVIII SHL	Emicizumab	
sevHA4	12	Yes, high	Yes	12 mos.	rFVIII SHL	psFVIII SHL, aPCC	
sevHA5	14	No	No	10 yrs.	psFVIII SHL	Emicizumab	Medication in country of origin unknown (immigrant)
sevHA6	16	No	Yes	2 yrs.	psFVIII SHL		
sevHA7	16	No	Yes	10 mos.	psFVIII SHL		
sevHA8	20	No	Yes	1 yr.	rFVIII SHL		
sevHA9	9	No	No	18 mos.	psFVIII SHL		Parents wanted reduced therapy
sevHA10	21	No	Yes	15 mos.	psFVIII SHL	Emicizumab	
sevHA11	1	Yes, high	Yes	11 mos.	Emicizumab		
sevHA12	14	No	Yes	4 yrs.	psFVIII SHL		
sevHA13	21	No	Yes	2 yrs.	psFVIII SHL	Emicizumab	
sevHA14	14	No	No	7 yrs.	psFVIII SHL		First therapy on demand, therapy not regularly
sevHA15	17	No	No	8 yrs.	psFVIII SHL		Joint damage at first visit (immigrant), non-compliance
sevHA16	18	No	No	6 yrs.	rFVIII SHL		Until 6 yrs. on demand
sevHA17	6	No	Yes	3 yrs.	rFVIII EHL	Emicizumab	
sevHA18	18	Yes, low	No	14 mos.	psFVIII SHL		Non-compliance
sevHA19	16	No	Yes	Unknown	rFVIII SHL		
sevHA20	18	Yes, low	Yes	11 mos.	rFVIII SHL		
sevHA21	10	No	Yes	14 mos.	psFVIII SHL		
sevHA22	20	No	No	7 yrs.	rFVIII SHL		Non-compliance
sevHA23	20	No	No	12 yrs.	psFVIII SHL		Non-compliance
mildHA24	16	No	On demand		rFVIII SHL		
modHA25	11	No	On demand		psFVIII SHL		
modHA26	15	No	On demand		psFVIII SHL		
modHA27	7	No	On demand		rFVIII SHL	Emicizumab	
modHA28	8	No	On demand		rFVIII SHL	Emicizumab	
modHA29	14	No	On demand		rFVIII SHL	Emicizumab	
modHA30	16	No	On demand		psFVIII SHL		
sevHB31	16	No	Yes	1 yr.	psFIX SHL		
sevHB32	18	No	Yes	18 mos.	psFIX SHL		
sevHB33	13	Yes, low	No	13 yrs.	rFIX SHL		Joint damage at first visit, medication in country of origin unknown (immigrant)
sevHB34	6	No	Yes	12 mos.	rFIX SHL		Immigrant
sevHB35	8	No	No	8 yrs.	rFIX SHL		Joint damage at first visit, medication in country of origin unknown (immigrant)
modHB36	7	No	On demand		rFIX EHL		
modHB37	12	No	On demand		psFIX SHL		
modHB38	15	No	On demand		psFIX SHL		
modHB39	19	No	On demand		psFIX SHL		
vWD40	10	Yes, low	Yes	Unknown	psFVIII SHL		Immigrant
vWD41	2	No	Yes	14 mos.	psFVIII SHL		Immigrant

HA, hemophilia A; HB, hemophilia B; vWD, von Willebrand disease; sev, severe; mod, moderate; mo., month; mos., months; yr., year; yrs., years; psFVIII, plasma derived FVIII; rFVIII, recombinant FVIII; SHL, standard half-life; EHL, extended half-life; aPCC, activated Prothrombin Complex Concentrate.

### 3.2 Assessment of MRIs applying the IPSG score

**FIGURE 1:**
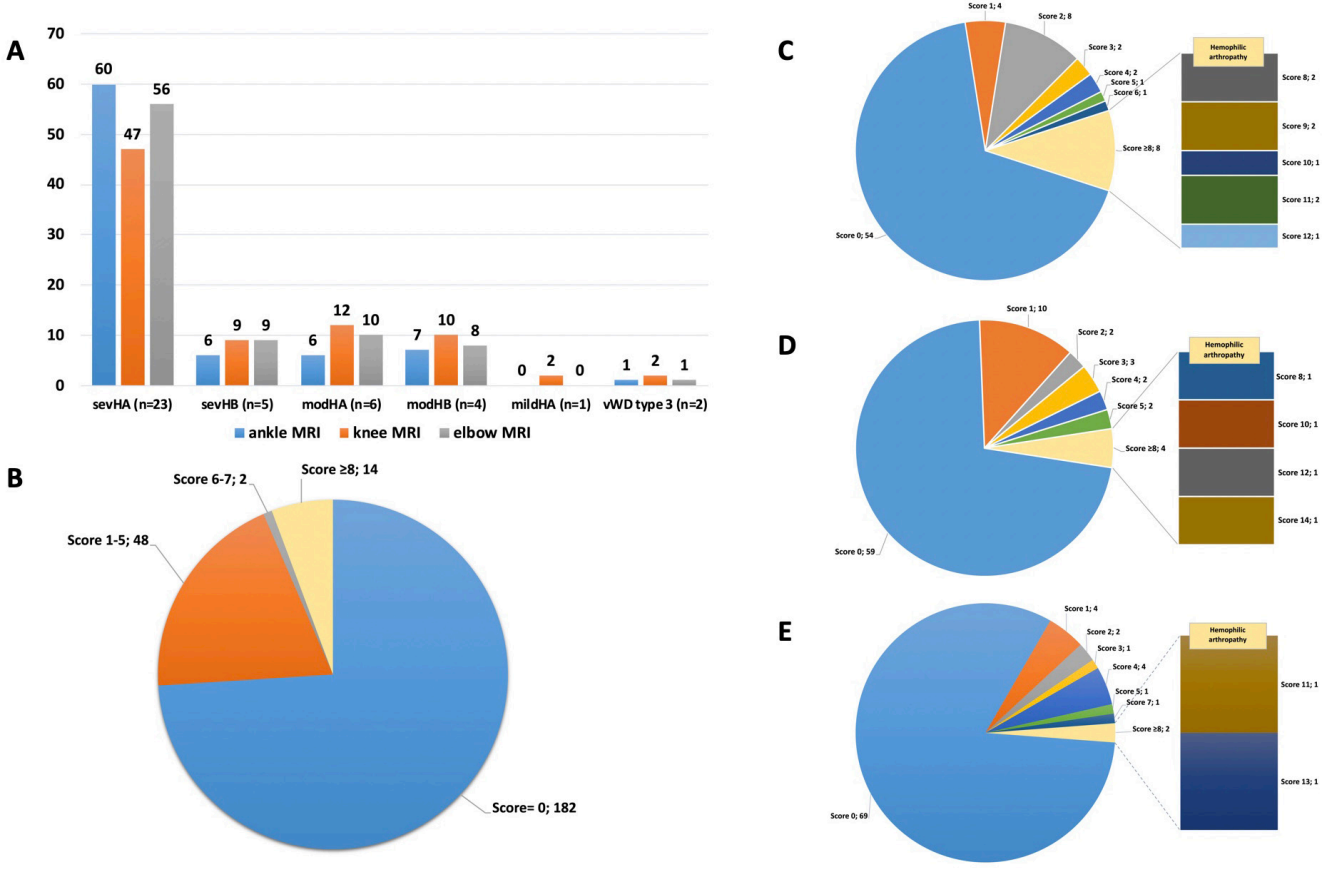
**(A)** Number of joint magnetic resonance imaging (MRIs) (*n* = 246) according to disease: sevHA/HB, patients with severe HA/HB; modHA/HB, patients with moderate HA/HB; mildHA, patient with mild HA; vWD, von Willebrand disease. **(B)** International Prophylaxis Study Group (IPSG) score distribution of the 246 MRI investigations. **(C)** Ankle joints (*n* = 80): Distribution of score 0–12. **(D)** Knee joints (*n* = 82): Distribution of score 0–14. **(E)** Elbow joints (*n* = 84): Distribution of score 0–13.

In total 246 MRIs had been performed for the participants of the study. The MRIs have been performed at different time points and also follow-up MRIs have been included. The majority of MRI examinations were performed in patients with sevHA. This group was the largest cohort of the study participants (23/41; 56%) and they underwent by far the most MRIs, particularly of the ankle (60/80), knee (47/82) and elbow (56/84) joints. In comparison, patients with sevHB and vWD type 3 underwent fewer MRIs, which is also reflected in their lower numbers in those groups. In the 6 patients with modHA and in the 4 patients with modHB a similar number of MRIs were performed (Figure 1A). A total of 14 (5.7%) of 246 joint MRIs showed severe joint changes (IPSG score ≥ 8). A total of 2 (0.81%) showed moderate alterations (IPSG score 6–7), 48 (19.5%) mild alterations (IPSG score 1–5), and 182 (73.9%) no alterations (IPSG score = 0) (Figure 1B). In our cohort, an IPSG score of ≥8 was consistent with the existing MRI reports indicating hemophilic arthropathy. This threshold was established by comparing the existing radiological reports at the time of MRI with the IPSG scores assigned by an independent radiologist.

### 3.3 MRI results per joint

#### 3.3.1 Ankle joints

A total of 54 (67.5%) of joints showed no changes in 80 MRI examinations of the ankle joints, thus achieving an IPSG score of 0. We observed minor changes in 17 (21.3%) of the ankle joints (IPSG score 1–5). Only 1 (1.3%) of ankle joint showed moderate damage (IPSG score 6–7). Finally, 8 (10.0%) of the ankle joints revealed advanced hemophilia arthropathy (IPSG score ≥ 8) (Figure 1C). Most of the ankle joints revealed no or only mild changes. Compared to the knee and elbow joints the ankle joints exhibit the most pathologies and arthropathies.

#### 3.3.2 Knee joints

A total of 82 MRIs of the knee joints revealed that 59 (72.0%) of the joints had no pathologies (IPSG score of 0). 19 (23.2%) of the knee joints showed mild damage (IPSG score 1–5). No joint (0%) showed moderate damage (IPSG score 6–7). Finally, 4 (4.9%) of the knee joints revealed advanced hemophilia arthropathy (IPSG score ≥ 8) (Figure 1D). These results indicate that the majority of knee joints presented no or only minor damage. The knee joint is the second most frequently affected joint in this study.

#### 3.3.3 Elbow joints

In 84 MRI examinations of the elbow joints, 69 (82.1%) showed no changes (IPSG score 0). Twelve (14.3%) of the elbow joints showed minor changes (IPSG score 1–5). One (1.2%) of the joints presented moderate changes (IPSG score 6–7). Finally, 2 (2.4%) of the elbow joints showed advanced hemophilia arthropathy (IPSG score ≥ 8) (Figure 1E). The majority of elbow joints revealed no or minimal joint alterations. The elbow joint is the least affected joint according to our study’s data.

Detailed information about the distribution of osteochondral and soft tissue subcategories in ankle, knee, and elbow joints are given in Supplementary Table 1 and Supplementary Figures 1–3, respectively.

### 3.4 Presentation of patients with inhibitors

A total of 7/41 (17.1%) patients (HA *n* = 5; HB *n* = 1; vWD *n* = 1) developed inhibitors against factor VIII or factor IX or vWF during the disease course. 3 of the 7 inhibitor patients (42.9%) presented with an intron 22 inversion (*F8* gene), which significantly increases the risk of developing inhibitors. Only one of the 3 patients with intron 22 inversion and inhibitors developed hemophilic arthropathy (33%).

In 2/7 (28.6%) patients with inhibitors hemophilic arthropathy was diagnosed, one of these two patients suffered from HB and had already developed arthropathy before he presented at our outpatient clinic. Both were successfully treated with ITT (within 12 months), however, in the subsequent disease course one patient (with HA) denied almost completely the regular, prophylactic FVIII-substitutions. Among patients with severe hemophilia, inhibitor presence showed no significant association with severe joint damage. Two out of six hemophilia patients (33%) with inhibitors had IPSG scores ≥ 8, compared to 5 out of 22 hemophilia patients (23%) without inhibitors (Fisher’s exact test, OR = 1.7, *p* = 0.622).

### 3.5 Presentation of patients with hemophilic arthropathy

**FIGURE 2:**
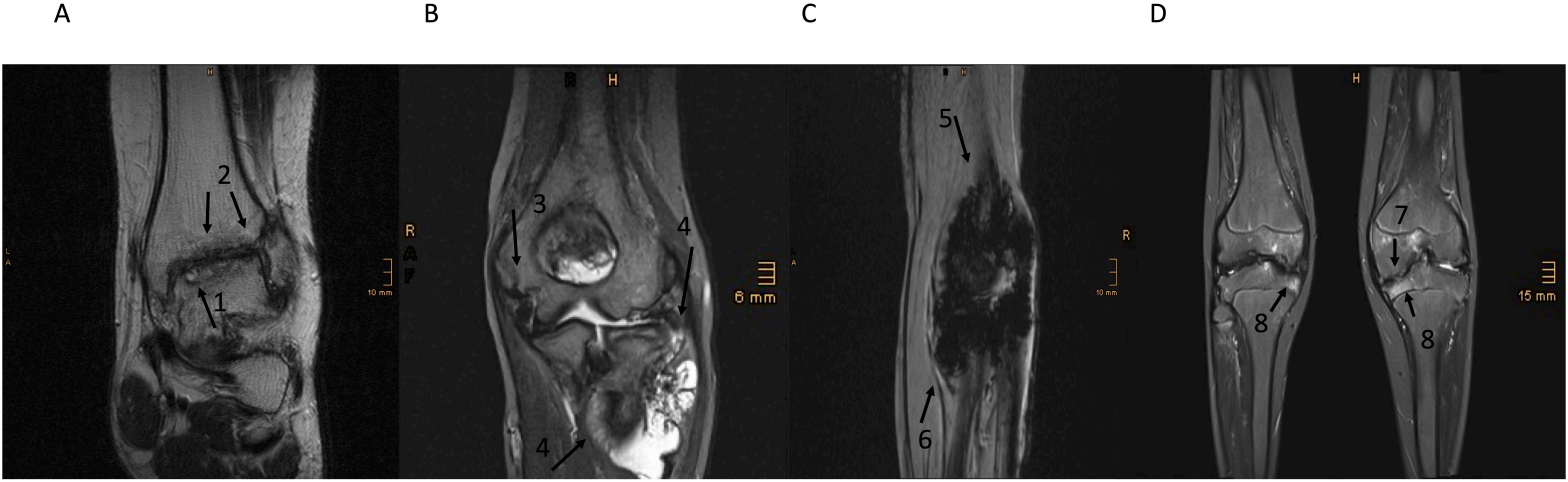
MRIs from patients with an IPSG score ≥ 8. **(A)** Left ankle pt. ID sevHA15 (coronal T2-weighted turbo spin echo): (1) subchondral cysts and (2) reduced joint space and joint incongruence. **(B)** Left elbow pt. ID sevHB35 (coronal T2-weighted turbo inversion recovery): (3) osseous joint destruction and (4) mass synovial hypertrophy with hemorrhagic parts. **(C)** Right elbow pt. ID sevHB35 (sagittal T2-weighted): (5, 6) pronounced hemosiderin artifacts appearing as dark or low-signal-intensity areas. **(D)** Knees pt. ID sevHB33 (coronal T2-weighted turbo inversion recovery): (7) osteophyts appearing as bony outgrowths, osseous joint destruction and joint incongruence, (8) bone marrow edema showing high signal on T2 weighted images.

A total of 7/41 (17.1%) patients suffered from hemophilic arthropathy (IPSG Score ≥ 8) indicated by soft tissue (e.g., synovial hypertrophy, Figure 2B; hemosiderin deposition, Figure 2C) and osteochondral pathologies (e.g., subchondral cyst, cartilage destruction, Figures 2A, D). All patients with IPSG scores ≥ 8 had severe hemophilia (7/28, 25%), compared to none in the moderate/mild group (0/11, 0%), though this trend was not statistically significant (Fisher’s exact test, *p* = 0.159).

The majority of patients 6/7 (85,7%) presenting hemophilic arthropathy (4 sevHA and 2 sevHB) did not receive primary prophylaxis for different reasons. Four immigrants had only received factor treatment on demand in their home country and presented with hemophilic arthropathy at the initial visit. Three patients were non-compliant regarding their therapy.

An IPSG score ≥ 8 indicates that joint damage is constant or progressive over time. In the three patients with hemophilic arthropathy (IPSG score ≥ 8) who underwent MRI follow-up exams, three showed a progressive course via a higher IPSG score. The third patient revealed a progressing disease with a higher IPSG score in one joint, while the other joint remained constant.

### 3.6 Presentation of patients with normal imaging data (IPSG score = 0)

Fifteen of 41 patients (9 sevHA, 1 modHA, 1 sevHB, 3 modHB, 1 vWD type 3) showed no changes (IPSG score 0) in their MRI examinations of the elbow, knee and upper ankle joint. Four of these patients had developed anti-FVIII antibodies and were treated successfully.

### 3.7 Presentation of patients with abnormal imaging data (IPSG score > 0 and < 8)

International Prophylaxis Study Group scores of ≤7 predominantly reflect soft tissue changes which can be reversible. A total of 10/28 of the patients with severe HA/HB had IPSG scores of 1–5 for their joints. These mild abnormalities were due to delayed therapy, non-compliance, sport, trauma or the patients preference for therapy on demand. One patient developed inhibitors during the treatment course. Only one patient with sevHA was once assigned an IPSG score of 7 due to recurrent hemorrhages caused by delayed treatment; this score fortunately decreased over time. 6 patients with moderate HA/HB, one with mild HA, and one with vWD type 3 had IPSG scores of 1–5 due to trauma, fracture, and increased physical activity/competitive sports.

In nine patients with an IPSG score > 0 < 8 who received an MRI follow-up, eight patients (4 sevHA, 1 sevHB, 2 modHA, 1 mildHA) showed an improvement with a decreasing IPSG score and one patient with sevHA had consistent scores. Of those with an IPSG score ≤ 3, all but one patient presented only soft tissue changes.

**FIGURE 3:**
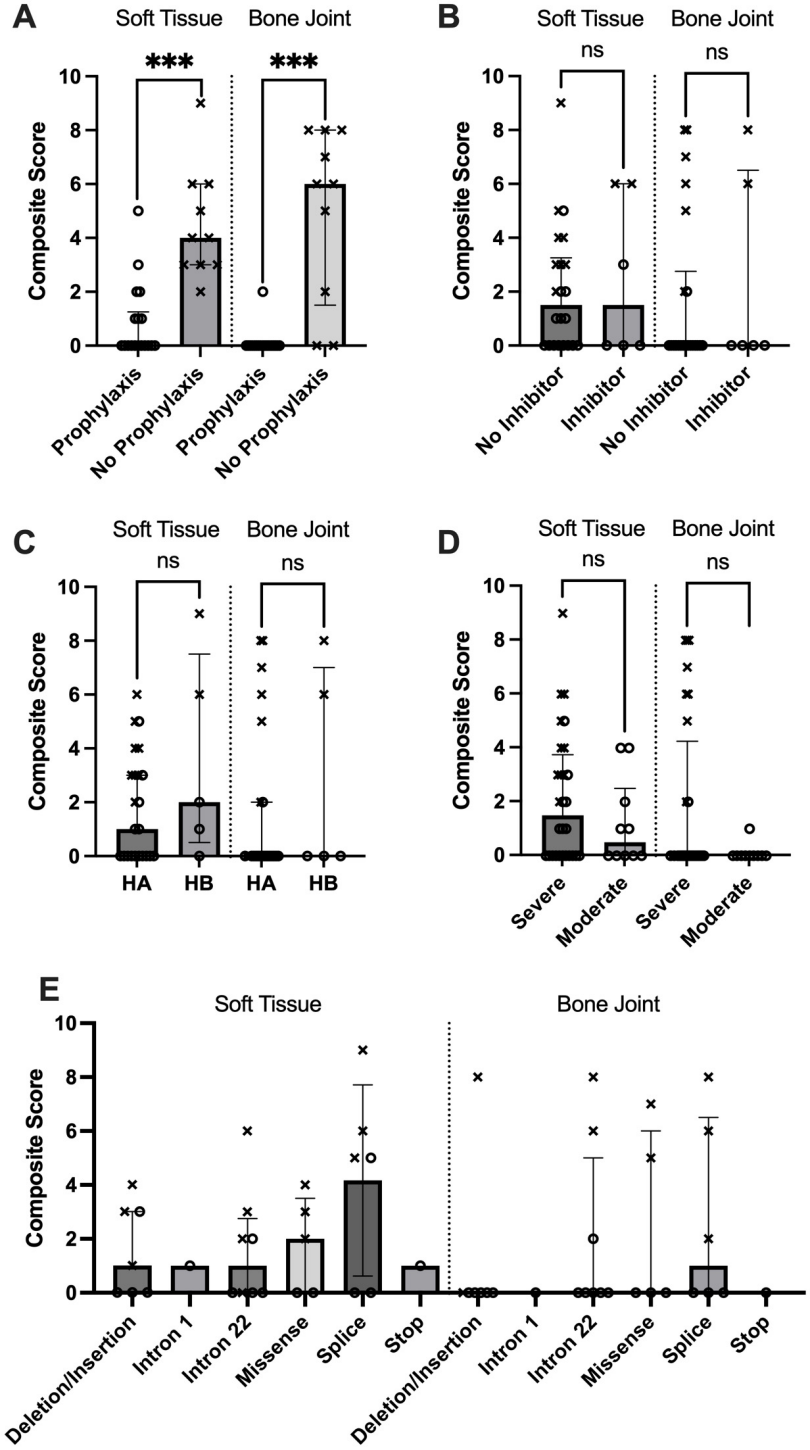
Maximum International Prophylaxis Study Group (IPSG) scores compared between patient groups. **(A–C)** Show data exclusively from patients with severe hemophilia, **(D,E)** include all hemophilia patients. Patients without continuous prophylaxis are marked with “x” symbols. Statistical significance was determined using the Mann-Whitney U test, with ***P < 0.001 [for panels **A–D)**].

In total 18 hemophilic patients received regular prophylaxis. All started prophylaxis between 10 months and 3 years of age except one patient who had been diagnosed late in another hospital and therefore, he started at age 4 years. Regular prophylaxis was strongly associated with reduced joint damage in severe hemophilia patients. No patient on prophylaxis (0/18) had a IPSG score ≥ 8, compared to 70% (7/10) of patients without regular prophylaxis (Fisher’s exact test, *p* < 0.001). The maximum IPSG score for each individual on a per-patient basis showed a statistically significant impact of prophylaxis using the Mann-Whitney U test (Figure 3A).

In comparisons stratified by hemophilia type, disease severity, and inhibitor status, no significant differences were found in either maximum IPSG component scores or the proportion of measurements with scores 0 (Figures 3B–D)

### 3.8 Molecular genetics

**FIGURE 4:**
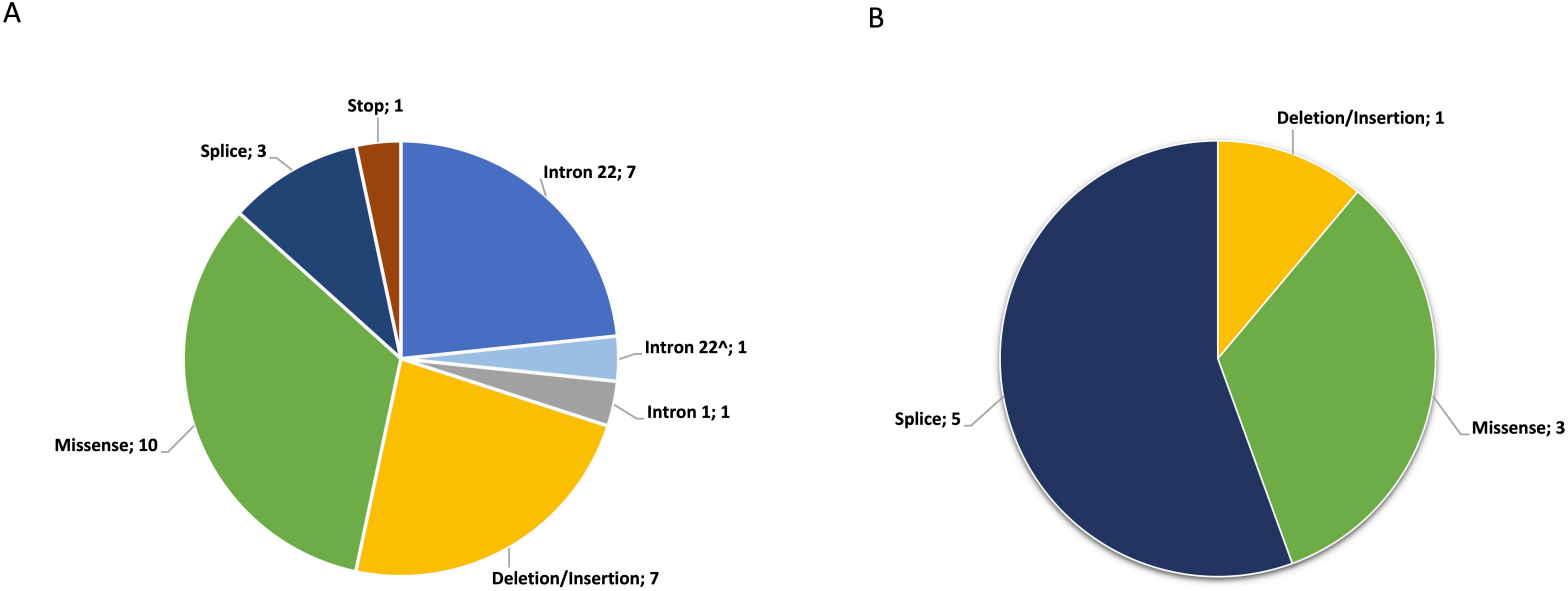
**(A)** Distribution of genetic alterations in patients with HA (*n* = 30); Intron 22^∧^, intron 22 inversion suspected in a patient with sevHA due to family history of intron 22 inversion (affected brother had been genetically investigated and had an intron 22 inversion). **(B)** Distribution of genetic alterations in patients with hemophilia B (HB) (*n* = 9)

The available molecular genetic reports revealed a broad spectrum of genetic alterations in patients with HA, HB and vWD in the associated genes *F8*, *F9*, and *VWF*, respectively. Loss of function mutations (intron-inversion, stop mutations, frameshift, splice defects) are associated with severe forms of the diseases (sHA 19/23; sHB 4/5; vWD type 3 2/2). For one patient (patient ID sevHB31 in Supplementary Table 3) a reclassification was not possible due to a lack of information in the original report (from 2005). Nevertheless, the original mutation (F9 exon deletion f-h) was included in Figure 4 as deletion/insertion finding. Genetic revaluation according to ACMG guidelines is available in Supplementary Tables 2–5.

An intron 22 inversion occurred in a total of 7/30 (23%) HA patients and presumably in one affected male (intron 22^∧^) due to the family history (an intron 22 inversion already had been diagnosed in his brother). An intron-1 inversion was present in one HA patient. Deletions and insertions were detected in eight patients (HA *n* = 7; HB *n* = 1). Splice site mutations affected eight patients (HA *n* = 3; HB *n* = 5). Two canonical splice site mutations were present in F8 (in three patients with severe HA) and F9 (in three patients with severe HB). The F9 c.520 + 13A > C mutation is known to have a moderate to mild phenotype, suggesting that this mutation leads to alternative splicing of various extent. A stop mutation was detected in one HA patient. The severity of these alterations was triggering a loss of function in either FVIII or FIX, and correlated with severe HA or HB. Missense mutations represented the largest group with 13 cases (HA *n* = 10; HB *n* = 3) and were found predominantly in patients with moderate/mild HA or HB. Individual composite IPSG scores stratified by mutation are shown in Figure 3E.

A homozygous frameshift mutation (c.3285_3307delinsTCC; p.Asp1096Profs*39) was detected in both VWD type 3 patients leading to loss of function of the vWF and severely reduced FVIII activity (2.5% and 3%, respectively).

## 4 Discussion

In this retrospective study we investigated 41 patients with HA, HB and von vWD type 3. With a share of 73%, HA was the most common diagnosis in our cohort. The severe forms predominated in HA (56% sevHA versus 12% sevHB). This aligns with the known epidemiological distribution (13). The prevalence of severe HA matches international data (10), the lower rate of severe HB cases may reflect the reduced mutational burden of F9 gene (13).

This study underscores the necessity of early, individualized prophylaxis to prevent hemophilic arthropathies. Our results are consistent with published data by Oldenburg et al. (10) who showed that initiating prophylaxis early reduces the risk of developing hemophilic arthropathies. Of the 28 patients with severe HA/HB who received early primary prophylaxis in our cohort, only one (3.6%) was non-compliant and was observed to have both arthropathy and inhibitor development Our results show that 67.5%–82.1% of the three joints (ankle 67.5%, knee 72%, and elbow 82.1%), examined in patients undergoing prophylactic substitution therapy remained unaffected (IPSG 0), even in the four patients who developed inhibitors during the treatment course. Reasons for this good outcome may be early start of individually adapted prophylaxis and patient education regarding the disease after identifying the diagnosis.

In addition, this retrospective study shows that although prophylactic substitution therapies are effective, they cannot always entirely prevent subclinical joint bleeding and changes, which underlines the need for comprehensive diagnostics (including MRI and/or ultrasound analyses). In our study, subclinical joint pathologies were observed in 14.3%–23.2% of MRIs (IPSG score 1–5) and were fortunately reversible and limited to soft tissue impairment. On the one hand, the causes thereof were delayed initiation of treatment, lack of adherence to treatment, excessive or higher-risk sports (i.e., skiing, playing soccer), trauma, or the patient’s preference for on-demand treatment (in previous times). Mild joint changes were addressed through individualized adjustments of therapy (prophylactic or therapeutic) in response to increased bleeding episodes, combined with structured physiotherapy aimed at preserving joint function and preventing further progression of arthropathy. These joint changes could also imply silent microbleeds despite adequate treatment, a phenomenon also reported by Van Leeuwen et al. (14). Persistent subclinical bleeding thus indicates incomplete protection under standard prophylaxis, possibly due to pharmacokinetic fluctuations or local inflammation (8).

If this leads to repeated hemarthroses, an autocatalytic process of degenerative joint changes can be triggered characterized by iron deposits, inflammatory processes, and synovial proliferation, which MRI can reveal as hemosiderin deposits and synovial hyperthrophy (15). In our study, we also detected on MRIs synovial hypertrophy in 25% of the ankle joints, in 13.4% of the knee joints, and in 13.1% of the elbow joints. Hemosiderin deposits were also detected in 16% of the ankle MRIs, in 9.8% of the knee MRIs and in 10.7% of the elbow MRIs. The detection of synovitis on MRI also makes the joint more vulnerable - a mechanism also reported in recent studies (14), in which synovial hyperplasia and hemosiderin deposition were identified as the most common feature.

Compared to the HEAD-US score that focuses on soft tissue pathology and the Pettersson score that evaluates radiographic destruction (16), the IPSG score enables comprehensive assessment. The IPSG score determined in our study proved to be a sensitive tool for detecting the entire arthropathic spectrum - from early synovitis (25% of ankle joints) and hemosiderosis (16% of ankle joints) to late-stage destruction (IPSG score ≥ 8: 10% of ankle joints).

Our study demonstrates that irreversible hemophilic arthropathy, assessed based on the initial MRI reports, correlates exclusively with IPSG scores ≥ 8, while lower scores (especially < 8) are predominantly associated with reversible changes. Because these changes are reversible and often due to accidents, we would assign severe hemophilic arthropathy a higher IPSG score. Accordingly, we considered a score of ≥8 as indicative of hemophilic arthropathy. As the use of the IPSG score in assessing joint health is still relatively uncommon and the existing literature is limited, no universally defined thresholds have yet been established.

The ankle joints were most often affected by hemophilic arthropathy in our study. This is consistent with previous studies showing that ankle joints are more susceptible to hemophilic arthropathy (9, 17).

Our study investigations also reveal a clear pattern in patients with advanced hemophilic arthropathy (IPSG score ≥ 8), all of whom did not have access to primary prophylaxis, started prophylaxis late, or failed to comply with prophylaxis. These problems were often associated with a delayed diagnosis or inadequate medical infrastructure in their home countries, particularly for the immigrants (4/7 patients with hemophilic arthropathy IPSG score ≥ 8), who often presented joint damage on their first visit to our hospital. This evidence underscores the need to improve early detection and equitable access to care, especially in regions with limited resources for hemophilia management.

Witkop et al. (18) reported that socioeconomic stress factors can impede therapy adherence, particularly psychosocial stress, which should be better addressed within patient care. Three of the seven patients with hemophilic arthropathy in this study failed to adhere to their therapy with the consequence of an IPSG score ≥ 8. At least 2/3 of patients not consistently adhering to their treatment suffered from socioeconomic stress factors (e.g., parents’ divorce, alcoholic father, parents’ unemployment). The burden of socioeconomic stress factors and the disease may have been too great for them, so that even regular counseling by a psychologist could not improve adherence.

The distribution of genetic mutations in our study reflects the genetic heterogeneity of these coagulopathies. As already reported in the literature (11), our study also shows that the same mutations can lead to different bleeding phenotypes i.e., the frequency of hemophilic arthropathy and development of factor inhibitors can also vary with the same underlying mutation.

An example is the intron 22 inversion considered a decisive risk factor for the development of inhibitors (19) and thus also raising the risk of developing hemophilia arthropathy: However, we could not confirm this as being significant for developing hemophilic arthropathy in our cohort only 2 of 6 hemophilia patients with inhibitors showed an IPSG score of ≥8 and showed signs of hemophilic arthropathy (MRI, clinical). Other studies revealed that patients with inhibitor showed more often signs of hemophiliac arthropathy (11). Only 3/7 (42.9%) of patients who developed inhibitors presented this inversion and only 2/7 (28.6%) patients diagnosed with hemophilic arthropathy revealed an inversion of intron 22. Interestingly, 4/7 patients who developed inhibitors even had the lowest IPSG score 0.

In summary, this study shows that the IPSG score is useful for assessing joint health in patients with hemophilia or vWD. The prophylactic therapy (with trough levels of >1%, or later >3%–5%) may have prevented a severe hemophilic arthropathy, however, was not sufficient to prevent mild soft tissue changes in these patients. Early diagnosis and primary prophylaxis as well as therapy adherence was associated with reduced joint damage.

## 5 Limitations

Our findings have several limitations to be considered. The retrospective collection of data carries the risk of incomplete documentation. The evaluation was conducted retrospectively and compared to the original clinical MRI reports from the time of imaging. The original MRI assessments were performed by a very experienced radiologist regarding pediatrics and joint diseases. As the purpose of the reassessment was to systematically apply the IPSG scoring system to historical data, no second independent reading for the score was performed. The independent radiologist assessing the scores was blinded regarding the severity of the disease and the therapy. In addition, the heterogeneity of the MRI protocols (e.g., missing hemosiderin-sensitive sequences in eight ankle joint and nine knee MRIs) underlines the need for standardized protocols.

Additionally, the study population from one single center was investigated which may introduce selection bias and limit the generalizability. Since this is a long-term study, some therapeutic changes have occurred during the time of the study. However, most of the patients have been treated with a Standard Half-Life (SHL) product three times a week (hemophilia A patients) or two times a week (hemophilia B patients). Only recently (between 2018 and 2023) 10 patients (7 sHA, 3 modHA) have started with the bispecific, factor VIII mimetic antibody therapy.

Regarding the statistical analysis performed for the cohort of hemophilic patients (*n* = 39), this study is limited by the small sample size with only seven patients presenting IPSG scores ≥ 8, resulting in limited statistical power and precluding multivariate analysis.

## 6 Conclusion

A total of 17.1% of our study patients with severe hemophilia were suffering from advanced arthropathy (IPSG score ≥ 8). This may be due to the fact that they did not have access to effective therapy such as factor prophylaxis, or failed to adhere to their therapy. Fortunately, none of our other study patients who adhered to the prophylactical treatment developed irreversible joint arthropathy – which underlines the potential benefits of consistent prophylaxis and adherence to therapy.

Overall, these findings underscore the importance of early diagnosis, continuous monitoring, and adherence to treatment in patients with these congenital coagulopathies. Early changes in joints can be detected by relying on the IPSG score before the devastating effects of hemophilic arthropathy become irreversible. Further prospective, multicenter studies using standardized protocols are warranted to validate these observations and better understand the long-term impact of prophylactic treatment strategies.

## Data Availability

The original contributions presented in this study are included in this article/Supplementary material, further inquiries can be directed to the corresponding author.
