# Supplementary material for: Impact of prophylaxis, inhibitors, and genetics on joint outcomes according to the IPSG-MRI score in hemophilia A, B and vWD type 3
Source: Front Med (Lausanne). 2026 Jan 27;12:1668012. doi: 10.3389/fmed.2025.1668012 (PMC12888774; doi:10.3389/fmed.2025.1668012)

# Supplementary Tables

Table S1: IPSG scores regarding soft tissue and osteochondral changes for the various joints at different time dates

L, left; R, right; BL, bilateral; xxx, hemosiderin-sensitive sequence was not performed

| **Pt.-ID** | **Joint MRI** | **Date of MRI**  **(MM/DD/YYYY)** | **SoftTissue** | | | **Osteochondral changes** | | | **Total score** |
| --- | --- | --- | --- | --- | --- | --- | --- | --- | --- |
|  |  |  | **Effusion/haemarthrosis (1-3)** | **Synovial hypertrophy (1-3)** | **Haemosiderin (1-3)** | **Surface erosions (1-2)** | **Subchondral cysts (1-2)** | **Cartilage degradation (1-4)** |  |
| sevHA1 | ankle L | 03/10/2018 | 2 | 2 | xxx | 0 | 0 | 0 | **4** |
|  | knee BL | 01/18/2021 | 0 | 0 | 0 | 0 | 0 | 0 | **0** |
|  | elbow R | 02/08/2021 | 0 | 0 | 0 | 0 | 0 | 0 | **0** |
|  | elbow L | 02/08/2021 | 0 | 0 | 0 | 0 | 0 | 0 | **0** |
|  | ankle BL | 02/10/2021 | 0 | 0 | 0 | 0 | 0 | 0 | **0** |
| sevHA2 | ankle BL | 05/24/2017 | 0 | 0 | 0 | 0 | 0 | 0 | **0** |
|  | knee BL | 09/04/2017 | 0 | 0 | 0 | 0 | 0 | 0 | **0** |
|  | elbow R | 09/08/2017 | 0 | 0 | 0 | 0 | 0 | 0 | **0** |
|  | ankle BL | 05/15/2019 | 0 | 0 | 0 | 0 | 0 | 0 | **0** |
| sevHA3 | knee R | 09/15/2015 | 0 | 0 | xxx | 0 | 0 | 0 | **0** |
|  | ankle BL | 11/07/2018 | 0 | 0 | 0 | 0 | 0 | 0 | **0** |
|  | knee BL | 11/17/2018 | 0 | 0 | 0 | 0 | 0 | 0 | **0** |
|  | elbow R | 11/21/2018 | 0 | 0 | 0 | 0 | 0 | 0 | **0** |
|  | elbow L | 11/28/2018 | 0 | 0 | 0 | 0 | 0 | 0 | **0** |
| sevHA4 | knee BL | 05/22/2017 | 0 | 0 | 0 | 0 | 0 | 0 | **0** |
|  | ankle BL | 03/06/2019 | 0 | 0 | 0 | 0 | 0 | 0 | **0** |
| sevHA5 | knee R | 02/28/2017 | 0 | 0 | xxx | 0 | 0 | 0 | **0** |
|  | ankle R | 03/29/2017 | 1 | 2 | xxx | 1 | 0 | 1 | **5** |
|  | ankle L | 03/29/2017 | 1 | 0 | xxx | 0 | 0 | 0 | **1** |
|  | knee L | 04/05/2017 | 1 | 0 | xxx | 0 | 0 | 0 | **1** |
|  | knee BL | 06/17/2019 | 0 | 0 | 0 | 0 | 0 | 0 | **0** |
|  | ankle R | 06/17/2019 | 0 | 1 | 2 | 1 | 1 | 1 | **6** |
|  | ankle L | 06/17/2019 | 1 | 1 | 0 | 0 | 0 | 0 | **2** |
|  | ankle R | 11/02/2020 | 1 | 1 | 2 | 1 | 2 | 2 | **9** |
|  | ankle L | 11/02/2020 | 1 | 0 | 0 | 0 | 0 | 0 | **1** |
| sevHA6 | knee BL | 09/03/2008 | 0 | 0 | 0 | 0 | 0 | 0 | **0** |
|  | ankle BL | 01/19/2009 | 1 | 0 | 0 | 0 | 0 | 0 | **1** |
|  | elbow R | 08/07/2009 | 0 | 0 | 0 | 0 | 0 | 0 | **0** |
|  | elbow L | 08/10/2009 | 0 | 0 | 0 | 0 | 0 | 0 | **0** |
|  | ankle BL | 07/01/2016 | 0 | 0 | 0 | 0 | 0 | 0 | **0** |
|  | knee BL | 07/06/2016 | 0 | 0 | 0 | 0 | 0 | 0 | **0** |
|  | elbow L | 07/11/2016 | 0 | 0 | 0 | 0 | 0 | 0 | **0** |
|  | elbow R | 08/01/2016 | 0 | 0 | 0 | 0 | 0 | 0 | **0** |
|  | elbow R | 07/06/2020 | 0 | 0 | 0 | 0 | 0 | 0 | **0** |
|  | elbow L | 07/08/2020 | 0 | 0 | 0 | 0 | 0 | 0 | **0** |
|  | knee BL | 07/20/2020 | 0 | 0 | 0 | 0 | 0 | 0 | **0** |
|  | ankle BL | 07/27/2020 | 0 | 0 | 0 | 0 | 0 | 0 | **0** |
| sevHA7 | ankle BL | 12/20/2010 | 0 | 0 | 0 | 0 | 0 | 0 | **0** |
|  | knee BL | 02/02/2011 | 0 | 0 | 0 | 0 | 0 | 0 | **0** |
|  | elbow R | 02/07/2011 | 0 | 0 | 0 | 0 | 0 | 0 | **0** |
|  | elbow L | 02/14/2011 | 0 | 0 | 0 | 0 | 0 | 0 | **0** |
|  | ankle BL | 11/30/2015 | 0 | 0 | 0 | 0 | 0 | 0 | **0** |
|  | knee BL | 12/21/2015 | 0 | 0 | 0 | 0 | 0 | 0 | **0** |
|  | elbow R | 02/22/2016 | 0 | 0 | 0 | 0 | 0 | 0 | **0** |
|  | elbow L | 02/29/2016 | 0 | 0 | 0 | 0 | 0 | 0 | **0** |
|  | ankle L | 07/11/2016 | 0 | 0 | 0 | 0 | 0 | 0 | **0** |
|  | ankle BL | 01/09/2017 | 0 | 0 | 0 | 0 | 0 | 0 | **0** |
|  | knee BL | 08/07/2020 | 0 | 0 | 0 | 0 | 0 | 0 | **0** |
|  | elbow BL | 05/26/2021 | 0 | 0 | 0 | 0 | 0 | 0 | **0** |
|  | knee BL | 11/24/2021 | 0 | 0 | 0 | 0 | 0 | 0 | **0** |
|  | ankle BL | 11/24/2021 | 0 | 0 | 0 | 0 | 0 | 0 | **0** |
| sevHA8 | ankle BL | 07/09/2007 | 0 | 0 | 0 | 0 | 0 | 0 | **0** |
|  | knee BL | 07/14/2008 | 0 | 0 | 0 | 0 | 0 | 0 | **0** |
|  | elbow R | 07/22/2009 | 0 | 0 | 0 | 0 | 0 | 0 | **0** |
|  | elbow L | 02/15/2010 | 0 | 0 | 0 | 0 | 0 | 0 | **0** |
|  | ankle BL | 08/08/2014 | 0 | 0 | 0 | 0 | 0 | 0 | **0** |
|  | knee BL | 02/18/2015 | 0 | 0 | 0 | 0 | 0 | 0 | **0** |
|  | elbow R | 02/27/2017 | 0 | 0 | 0 | 0 | 0 | 0 | **0** |
|  | elbow L | 04/10/2017 | 0 | 0 | 0 | 0 | 0 | 0 | **0** |
|  | knees BL | 04/15/2019 | 0 | 0 | 0 | 0 | 0 | 0 | **0** |
|  | ankle BL | 04/17/2019 | 0 | 0 | 0 | 0 | 0 | 0 | **0** |
|  | elbow L | 04/26/2022 | 0 | 0 | 0 | 0 | 0 | 0 | **0** |
|  | ankle R | 07/06/2022 | 0 | 0 | xxx | 0 | 0 | 0 | **0** |
|  | elbow R | 10/25/2022 | 0 | 0 | 0 | 0 | 0 | 0 | **0** |
| sevHA9 | elbow R | 11/02/2022 | 0 | 0 | 0 | 0 | 0 | 0 | **0** |
|  | elbow L | 11/04/2022 | 0 | 0 | 0 | 0 | 0 | 0 | **0** |
|  | ankle R | 11/09/2022 | 2 | 0 | 0 | 0 | 0 | 0 | **2** |
|  | ankle L | 11/02/2022 | 0 | 0 | 0 | 0 | 0 | 0 | **0** |
|  | knee BL | 11/09/2022 | 0 | 0 | 0 | 0 | 0 | 0 | **0** |
| sevHA10 | knee BL | 10/16/2006 | 0 | 0 | 0 | 0 | 0 | 0 | **0** |
|  | ankle BL | 10/28/2009 | 0 | 0 | 0 | 0 | 0 | 0 | **0** |
|  | knee BL | 08/07/2013 | 0 | 0 | 0 | 0 | 0 | 0 | **0** |
|  | ankle BL | 08/07/2013 | 0 | 0 | 0 | 0 | 0 | 0 | **0** |
|  | knee BL | 02/14/2018 | 0 | 0 | 0 | 0 | 0 | 0 | **0** |
|  | ankle BL | 02/14/2018 | 0 | 0 | 0 | 0 | 0 | 0 | **0** |
|  | elbow L | 03/28/2018 | 0 | 0 | 0 | 0 | 0 | 0 | **0** |
|  | elbow R | 04/04/2018 | 0 | 0 | 0 | 0 | 0 | 0 | **0** |
|  | ankle R | 01/31/2023 | 0 | 0 | 0 | 0 | 0 | 0 | **0** |
|  | ankle L | 02/01/2023 | 0 | 0 | 0 | 0 | 0 | 0 | **0** |
|  | knee L | 03/31/2023 | 3 | 2 | 0 | 0 | 0 | 0 | **5** |
|  | elbow L | 08/01/2023 | 3 | 2 | 0 | 0 | 0 | 0 | **5** |
| sevHA11 | elbow R | 06/19/2023 | 1 | 2 | 0 | 0 | 0 | 0 | **3** |
| sevHA12 | ankle R | 10/29/2002 | 0 | 0 | xxx | 0 | 0 | 0 | **0** |
|  | knee L | 08/06/2003 | 1 | 0 | 0 | 0 | 0 | 0 | **1** |
|  | knee R | 08/12/2003 | 0 | 0 | 0 | 0 | 0 | 0 | **0** |
|  | ankle L | 06/03/2004 | 0 | 0 | xxx | 0 | 0 | 0 | **0** |
|  | knee BL | 08/24/2007 | 0 | 0 | 0 | 0 | 0 | 0 | **0** |
|  | elbow R | 01/07/2009 | 0 | 0 | 0 | 0 | 0 | 0 | **0** |
|  | elbow L | 04/15/2009 | 0 | 0 | 0 | 0 | 0 | 0 | **0** |
|  | ankle BL | 04/07/2010 | 1 | 0 | xxx | 0 | 0 | 0 | **1** |
| sevHA13 | ankle BL | 04/08/2004 | 0 | 0 | xxx | 0 | 0 | 0 | **0** |
|  | knee BL | 02/09/2007 | 0 | 0 | 0 | 0 | 0 | 0 | **0** |
|  | elbow R | 06/29/2009 | 0 | 0 | 0 | 0 | 0 | 0 | **0** |
|  | elbow L | 12/14/2009 | 0 | 0 | 0 | 0 | 0 | 0 | **0** |
|  | ankle R | 08/16/2017 | 0 | 0 | 0 | 0 | 0 | 0 | **0** |
|  | ankle L | 08/21/2017 | 0 | 0 | 0 | 0 | 0 | 0 | **0** |
|  | elbow R | 08/22/2017 | 0 | 0 | 0 | 0 | 0 | 0 | **0** |
|  | elbow L | 08/23/2017 | 0 | 0 | 0 | 0 | 0 | 0 | **0** |
| sevHA13 | elbow L | 05/17/2019 | 0 | 0 | 0 | 0 | 0 | 0 | **0** |
|  | ankle R | 09/11/2002 | 0 | 0 | 0 | 0 | 0 | 0 | **0** |
|  | ankle L | 12/02/2003 | 1 | 1 | 1 | 0 | 0 | 0 | **3** |
|  | knee BL | 03/28/2007 | 0 | 0 | 0 | 0 | 0 | 0 | **0** |
|  | ankle BL | 10/27/2008 | 0 | 1 | 1 | 0 | 0 | 0 | **2** |
|  | elbow R | 12/07/2009 | 1 | 1 | 0 | 0 | 0 | 0 | **2** |
|  | ankle L | 06/29/2011 | 1 | 1 | 1 | 0 | 0 | 0 | **3** |
| sevHA15 | ankle L | 02/26/2003 | 0 | 2 | 2 | 2 | 2 | 4 | **12** |
| sevHA16 | elbow L | 05/19/2005 | 1 | 2 | 2 | 1 | 1 | 0 | **7** |
|  | elbow R | 05/23/2005 | 0 | 0 | 0 | 0 | 0 | 0 | **0** |
|  | knee BL | 08/18/2005 | 0 | 0 | 0 | 0 | 0 | 0 | **0** |
|  | ankle BL | 06/21/2006 | 0 | 1 | 1 | 0 | 0 | 0 | **2** |
|  | elbow L | 03/04/2009 | 1 | 2 | 1 | 0 | 0 | 0 | **4** |
|  | elbow R | 10/07/2009 | 0 | 0 | 0 | 0 | 0 | 0 | **0** |
|  | knee BL | 03/03/2010 | 0 | 0 | 0 | 0 | 0 | 0 | **0** |
|  | ankle BL | 10/13/2010 | 0 | 0 | 0 | 0 | 0 | 0 | **0** |
|  | knee BL | 03/03/2015 | 0 | 0 | 0 | 0 | 0 | 0 | **0** |
|  | elbow L | 03/05/2015 | 0 | 1 | 1 | 0 | 0 | 0 | **2** |
|  | elbow R | 03/10/2015 | 0 | 0 | 1 | 0 | 0 | 0 | **1** |
| sevHA17 | ankle BL | 11/24/2021 | 0 | 0 | 0 | 0 | 0 | 0 | **0** |
| sevHA18 | knee L | 03/04/2003 | 1 | 0 | xxx | 0 | 0 | 0 | **1** |
|  | knee R | 03/05/2003 | 1 | 0 | 0 | 0 | 0 | 0 | **1** |
|  | ankle R | 03/11/2003 | 0 | 1 | 1 | 0 | 0 | 0 | **2** |
|  | ankle L | 03/13/2003 | 0 | 1 | 1 | 0 | 0 | 0 | **2** |
|  | knee BL | 09/12/2007 | 0 | 0 | 0 | 0 | 0 | 0 | **0** |
|  | ankle BL | 10/31/2007 | 0 | 1 | 1 | 0 | 0 | 0 | **2** |
|  | elbow R | 04/18/2008 | 0 | 0 | 0 | 0 | 0 | 0 | **0** |
|  | elbow L | 05/02/2008 | 0 | 0 | 0 | 0 | 0 | 0 | **0** |
|  | knee R | 07/09/2012 | 0 | 0 | 0 | 0 | 0 | 0 | **0** |
|  | knee L | 07/09/2012 | 1 | 1 | 1 | 0 | 0 | 0 | **3** |
|  | knee L | 03/27/2013 | 2 | 2 | 2 | 1 | 2 | 1 | **10** |
|  | knee R | 06/02/2014 | 2 | 1 | xxx | 0 | 0 | 0 | **3** |
|  | knee L | 07/02/2014 | 2 | 2 | 2 | 2 | 2 | 2 | **12** |
| sevHA19 | knee BL | 09/08/2010 | 0 | 0 | 0 | 0 | 0 | 0 | **0** |
|  | ankle BL | 09/10/2010 | 0 | 0 | 0 | 0 | 0 | 0 | **0** |
|  | elbow R | 09/15/2010 | 0 | 0 | 0 | 0 | 0 | 0 | **0** |
|  | elbow L | 09/22/2010 | 0 | 1 | 1 | 0 | 1 | 1 | **4** |
|  | elbow R | 03/16/2015 | 0 | 0 | 0 | 0 | 0 | 0 | **0** |
|  | elbow L | 03/23/2015 | 0 | 1 | 1 | 0 | 1 | 1 | **4** |
| sevHA20 | ankle BL | 09/15/2008 | 0 | 0 | 0 | 0 | 0 | 0 | **0** |
|  | elbow R | 08/25/2014 | 0 | 0 | 0 | 0 | 0 | 0 | **0** |
|  | elbow L | 01/28/2015 | 0 | 0 | 0 | 0 | 0 | 0 | **0** |
|  | knee BL | 07/24/2015 | 0 | 0 | 0 | 0 | 0 | 0 | **0** |
|  | ankle BL | 05/24/2017 | 0 | 0 | 0 | 0 | 0 | 0 | **0** |
|  | elbow L | 09/04/2017 | 0 | 0 | 0 | 0 | 0 | 0 | **0** |
|  | elbow R | 09/08/2017 | 0 | 0 | 0 | 0 | 0 | 0 | **0** |
| sevHA21 | ankle BL | 12/20/2006 | 0 | 0 | 0 | 0 | 0 | 0 | **0** |
|  | elbow R | 07/07/2010 | 0 | 0 | 0 | 0 | 0 | 0 | **0** |
|  | ankle BL | 07/13/2011 | 0 | 0 | 0 | 0 | 0 | 0 | **0** |
|  | knee BL | 07/20/2011 | 0 | 0 | 0 | 0 | 0 | 0 | **0** |
|  | elbow L | 07/27/2011 | 0 | 0 | 0 | 0 | 0 | 0 | **0** |
| sevHA22 | knee R | 10/26/2000 | 2 | 1 | 0 | 0 | 0 | 0 | **3** |
|  | knee R | 05/14/2003 | 0 | 0 | 0 | 0 | 0 | 0 | **0** |
|  | knee L | 05/19/2003 | 1 | 0 | 0 | 0 | 0 | 0 | **1** |
|  | ankle L | 05/20/2003 | 0 | 0 | 0 | 2 | 0 | 2 | **4** |
|  | ankle R | 05/21/2003 | 0 | 1 | 1 | 2 | 2 | 2 | **8** |
|  | knee BL | 09/14/2006 | 0 | 0 | 0 | 0 | 0 | 0 | **0** |
|  | ankle R | 09/26/2006 | 0 | 1 | 1 | 2 | 2 | 3 | **9** |
|  | ankle L | 09/26/2006 | 0 | 1 | 1 | 2 | 2 | 2 | **8** |
| sevHA23 | ankle R | 10/01/2003 | 1 | 1 | 1 | 2 | 2 | 4 | **11** |
|  | ankle L | 10/08/2003 | 0 | 1 | 1 | 2 | 2 | 4 | **10** |
|  | knee BL | 10/10/2005 | 0 | 0 | 0 | 0 | 0 | 0 | **0** |
|  | elbow R | 10/11/2005 | 0 | 0 | 0 | 0 | 0 | 0 | **0** |
|  | ankle BL | 08/15/2007 | 1 | 1 | 1 | 2 | 2 | 4 | **11** |
|  | elbow L | 08/20/2007 | 0 | 0 | 0 | 0 | 0 | 0 | **0** |
| mildHA24 | knee L | 02/11/2009 | 3 | 2 | xxx | 0 | 0 | 0 | **5** |
|  | knee L | 11/07/2016 | 1 | 0 | 1 | 0 | 0 | 0 | **2** |
| modHA25 | knee R | 04/24/2017 | 0 | 0 | 0 | 0 | 0 | 0 | **0** |
|  | knee L | 04/24/2017 | 1 | 0 | 0 | 0 | 0 | 0 | **1** |
|  | elbow R | 11/17/2017 | 0 | 0 | 0 | 0 | 0 | 0 | **0** |
|  | ankle BL | 02/28/2018 | 0 | 0 | 0 | 0 | 0 | 0 | **0** |
|  | elbow L | 03/07/2018 | 0 | 0 | 0 | 0 | 0 | 0 | **0** |
|  | knee R | 05/03/2021 | 0 | 0 | 0 | 0 | 0 | 0 | **0** |
|  | knee L | 05/03/2021 | 0 | 0 | 0 | 0 | 0 | 0 | **0** |
| modHA26 | ankle BL | 03/20/2023 | 0 | 0 | 0 | 0 | 0 | 0 | **0** |
|  | knee BL | 03/20/2023 | 0 | 0 | 0 | 0 | 0 | 0 | **0** |
|  | elbow L | 03/22/2023 | 0 | 0 | 0 | 0 | 0 | 0 | **0** |
|  | elbow R | 03/24/2023 | 0 | 0 | 0 | 0 | 1 | 0 | **1** |
| modHA27 | ankle BL | 08/12/2022 | 1 | 1 | 0 | 0 | 0 | 0 | **2** |
|  | knee BL | 09/12/2022 | 0 | 0 | 0 | 0 | 0 | 0 | **0** |
|  | elbow BL | 09/21/2022 | 0 | 0 | 0 | 0 | 0 | 0 | **0** |
| modHA28 | elbow R | 09/29/2021 | 0 | 0 | 0 | 0 | 0 | 0 | **0** |
|  | elbow L | 09/27/2021 | 0 | 0 | 0 | 0 | 0 | 0 | **0** |
|  | knee BL | 09/27/2021 | 0 | 0 | 0 | 0 | 0 | 0 | **0** |
|  | ankle BL | 09/24/2021 | 0 | 0 | 0 | 0 | 0 | 0 | **0** |
| modHA29 | ankle BL | 11/13/2017 | 0 | 0 | 0 | 0 | 0 | 0 | **0** |
|  | knee BL | 12/01/2017 | 1 | 0 | 0 | 0 | 0 | 0 | **1** |
|  | elbow R | 01/12/2018 | 0 | 0 | 0 | 0 | 0 | 0 | **0** |
|  | elbow L | 02/09/2018 | 0 | 0 | 0 | 0 | 0 | 0 | **0** |
|  | ankle BL | 09/24/2021 | 0 | 0 | 0 | 0 | 0 | 0 | **0** |
|  | knee BL | 09/29/2021 | 0 | 0 | 0 | 0 | 0 | 0 | **0** |
|  | elbow R | 02/24/2022 | 0 | 0 | 0 | 0 | 0 | 0 | **0** |
|  | knee R | 07/11/2023 | 0 | 0 | 0 | 0 | 0 | 0 | **0** |
| modHA30 | knee BL | 01/14/2008 | 0 | 0 | 0 | 0 | 0 | 0 | **0** |
|  | knee L | 02/19/2014 | 3 | 0 | 1 | 0 | 0 | 0 | **4** |
| sevHB31 | ankle BL | 10/27/2014 | 0 | 0 | 0 | 0 | 0 | 0 | **0** |
|  | knee BL | 07/29/2016 | 0 | 0 | 0 | 0 | 0 | 0 | **0** |
|  | knee BL | 10/05/2020 | 1 | 0 | 0 | 0 | 0 | 0 | **1** |
|  | ankle BL | 10/12/2020 | 0 | 0 | 0 | 0 | 0 | 0 | **0** |
|  | elbow R | 10/16/2020 | 1 | 0 | 0 | 0 | 0 | 0 | **1** |
|  | elbow L | 10/21/2020 | 1 | 0 | 0 | 0 | 0 | 0 | **1** |
| sevHB32 | knee BL | 10/21/2009 | 1 | 0 | 0 | 0 | 0 | 0 | **1** |
|  | ankle BL | 02/05/2014 | 0 | 0 | 0 | 0 | 0 | 0 | **0** |
|  | elbow R | 03/26/2014 | 0 | 0 | 0 | 0 | 0 | 0 | **0** |
|  | knee R | 01/26/2016 | 1 | 1 | 0 | 0 | 0 | 0 | **2** |
|  | elbow L | 02/15/2016 | 0 | 0 | 0 | 0 | 0 | 0 | **0** |
|  | ankle BL | 11/24/2021 | 0 | 0 | 0 | 0 | 0 | 0 | **0** |
|  | knee BL | 09/21/2022 | 1 | 0 | 0 | 0 | 0 | 0 | **1** |
|  | elbow R | 09/22/2022 | 0 | 0 | 0 | 0 | 0 | 0 | **0** |
| sevHB33 | knee BL | 08/11/2021 | 0 | 3 | 3 | 2 | 2 | 4 | **14** |
|  | elbow R | 08/13/2021 | 1 | 2 | 1 | 2 | 1 | 4 | **11** |
| sevHB34 | elbow BL | 10/22/2021 | 0 | 0 | 0 | 0 | 0 | 0 | **0** |
|  | knee BL | 11/08/2021 | 0 | 0 | 0 | 0 | 0 | 0 | **0** |
|  | ankle BL | 11/08/2021 | 0 | 0 | 0 | 0 | 0 | 0 | **0** |
| sevHB35 | elbow L | 10/01/2021 | 3 | 3 | 3 | 1 | 0 | 3 | **13** |
|  | elbow R | 10/22/2021 | 0 | 0 | 0 | 0 | 0 | 0 | **0** |
|  | knee R | 11/08/2021 | 0 | 0 | 0 | 0 | 0 | 0 | **0** |
|  | knee L | 11/08/2021 | 0 | 1 | 1 | 2 | 1 | 3 | **8** |
|  | ankle BL | 11/08/2021 | 0 | 0 | 0 | 0 | 0 | 0 | **0** |
| modHB36 | knee BL | 03/11/2020 | 0 | 0 | 0 | 0 | 0 | 0 | **0** |
|  | ankle BL | 09/22/2021 | 0 | 0 | 0 | 0 | 0 | 0 | **0** |
| modHB37 | ankle BL | 04/27/2015 | 0 | 0 | 0 | 0 | 0 | 0 | **0** |
|  | knee BL | 05/04/2015 | 0 | 0 | 0 | 0 | 0 | 0 | **0** |
|  | knee R | 08/09/2018 | 0 | 0 | xxx | 0 | 0 | 0 | **0** |
|  | knee L | 09/10/2018 | 0 | 0 | xxx | 0 | 0 | 0 | **0** |
|  | ankle BL | 10/20/2018 | 0 | 0 | 0 | 0 | 0 | 0 | **0** |
|  | elbow L | 11/03/2018 | 0 | 0 | 0 | 0 | 0 | 0 | **0** |
|  | elbow R | 12/15/2018 | 0 | 0 | 0 | 0 | 0 | 0 | **0** |
| modHB38 | knee R | 08/09/2018 | 0 | 0 | xxx | 0 | 0 | 0 | **0** |
|  | knee L | 09/10/2018 | 0 | 0 | 0 | 0 | 0 | 0 | **0** |
|  | ankle BL | 10/20/2018 | 0 | 0 | 0 | 0 | 0 | 0 | **0** |
|  | elbow L | 11/03/2018 | 0 | 0 | 0 | 0 | 0 | 0 | **0** |
|  | elbow R | 12/15/2018 | 0 | 0 | 0 | 0 | 0 | 0 | **0** |
| modHA30 | knee BL | 01/14/2008 | 0 | 0 | 0 | 0 | 0 | 0 | **0** |
|  | knee L | 02/19/2014 | 3 | 0 | 1 | 0 | 0 | 0 | **4** |
| modHB39 | knee BL | 07/15/2016 | 0 | 0 | 0 | 0 | 0 | 0 | **0** |
|  | elbow R | 07/20/2016 | 0 | 0 | 0 | 0 | 0 | 0 | **0** |
|  | elbow L | 07/29/2016 | 0 | 0 | 0 | 0 | 0 | 0 | **0** |
|  | knee R | 12/16/2016 | 0 | 0 | 0 | 0 | 0 | 0 | **0** |
|  | ankle BL | 03/08/2017 | 0 | 0 | 0 | 0 | 0 | 0 | **0** |
|  | knee R | 12/08/2021 | 0 | 0 | 0 | 0 | 0 | 0 | **0** |
|  | knee L | 12/09/2021 | 0 | 0 | 0 | 0 | 0 | 0 | **0** |
|  | ankle L | 05/09/2022 | 0 | 0 | 0 | 0 | 0 | 0 | **0** |
|  | elbow L | 05/17/2022 | 2 | 1 | 1 | 0 | 0 | 0 | **4** |
|  | elbow R | 05/18/2022 | 0 | 0 | 0 | 0 | 0 | 0 | **0** |
|  | ankle R | 06/01/2022 | 0 | 0 | 0 | 0 | 0 | 0 | **0** |
| vWD40 | ankle BL | 08/27/2021 | 0 | 0 | 0 | 0 | 0 | 0 | **0** |
|  | knee BL | 12/08/2021 | 0 | 0 | 0 | 0 | 0 | 0 | **0** |
|  | elbow BL | 03/11/2022 | 0 | 0 | 0 | 0 | 0 | 0 | **0** |
| vWD41 | knee L | 07/30/2018 | 2 | 1 | 1 | 0 | 0 | 0 | **4** |

## Table S2: Maximum IPSG component scores and proportion of measurements >0 in patients with and without prophylaxis.

| Component | With prophylaxis (Score) | Without prophylaxis  (Score) | P | With prophylaxis (%>0) | Without prophylaxis (%>0) | P (%>0) |
| --- | --- | --- | --- | --- | --- | --- |
| Effusion/Haemarthrosis | 0.00  (0.00-1.00) | 1.00  (1.00-2.00) | 0.011 | 0%  (0-19) | 29%  (20-49) | 0.011 |
| Synovial Hypertrophy | 0.00  (0.00-0.75) | 2.00  (1.00-2.00) | 0.001 | 0%  (0-9) | 50%  (41-63) | 0.000 |
| Haemosiderin | 0.00  (0.00-0.00) | 2.00  (1.00-2.00) | 0.000 | 0%  (0-0) | 46%  (38-50) | 0.000 |
| Surface Erosions | 0.00  (0.00-0.00) | 2.00  (1.00-2.00) | 0.000 | 0%  (0-0) | 37%  (11-50) | 0.000 |
| Subchondral Cysts | 0.00  (0.00-0.00) | 2.00  (1.00-2.00) | 0.000 | 0%  (0-0) | 21%  (11-47) | 0.000 |
| Cartilage Degradation | 0.00  (0.00-0.00) | 2.50  (0.50-3.75) | 0.000 | 0%  (0-0) | 37%  (4-50) | 0.000 |

Data are presented as median (IQR). Comparisons were performed using the Mann–Whitney U-test.

Table S3: Genetic revaluation for patients with HA

In original genetic reports protein position is often given as position in mature FVIII without signal peptide (minus 19 aa); revaluation using MANE Select Transcripts (NM_000132.4(F8); ^, suspected due to family history; n.a., not available; n.l., not listed; data base query 04/2025

| **ID** | **Original genetic information** | **Revaluation** | **Distribution of genetic alterations (Figure 3)** | **EAHAD** | **dbSNP v156** | **gnomAD v2.1.1** | **Clinvar** | **ACMG**  **classification** |
| --- | --- | --- | --- | --- | --- | --- | --- | --- |
| sevHA1 | Intron 22 inversion | Intron 22 inversion | Intron 22 |  |  |  |  | pathogenic |
| sevHA2 | n.a. | Intron 22 inversion^ | Intron 22^ |  |  |  |  | pathogenic |
| sevHA3 | c.1824_1825delGA | c.1824_1825delGA, p.Asn609Tyrfs*14 | Deletion/ Insertion | n.l. | n.l. | n.l. | n.l. | pathogenic |
| sevHA4 | Intron 22 inversion | Intron 22 inversion | Intron 22 |  |  |  |  | pathogenic |
| sevHA5 | c.901C>T, p.Arg301Cys | c.901C>T, p.Arg301Cys | Missense | 32 patients reported, from mild to severe, mostly severe HA | rs1401805753 | n.l. | ID 1684376 | pathogenic |
| sevHA6 | Intron 1 inversion | Intron 1 inversion | Intron 1 |  |  |  |  | pathogenic |
| sevHA7 | c.5252A>G, p.Lys1732Arg | c.5252A>G, p.Lys1751Arg | Missense | 3 patients reported severe HA | n.l. | n.l. | n.l. | pathogenic |
| sevHA8 | Exon 1-6 deletion | Exon 1-6 deletion | Deletion/ Insertion |  |  |  |  | pathogenic |
| sevHA9 | Intron 22 inversion | Intron 22 inversion | Intron 22 |  |  |  |  | pathogenic |
| sevHA10 | IVS 15+1(G>A) | c.5373+1G>A | Splice | 5 patients reported with severe HA | rs1387946073 | n.l. | n.l. | pathogenic |
| sevHA11 | c.1737_1743delTCCAAGA, p.Asp579Glu*Ter5 | c.1737_1743delTCCAAGA, p.Asp579Glu*Ter5 | Deletion/ Insertion | n.l. | n.l. | n.l. | n.l. | pathogenic |
| sevHA12 | Arg(CGA)427Stop(TGA) | c.1336C>T p.Arg446* | Stop | 33 patients reported with severe HA | rs137852372 | n.l. | ID 10145 | pathogenic |
| sevHA13 | Gly(GGG)520 Trp(TGG) (mature FVIII) | c.1615G>T p.Gly539Trp | Missense | not listed (amino acid position listed with Gly539Glu reported 6 patients with moderate to mild) | n.l. | n.l. | n.l. | likely pathogenic |
| sevHA14 | Exon 14 Del A codons 1191 bis 1194 | c.3637del  p. Ile1213Phefs*5 | Deletion/ Insertion | 202 patients reported with severe HA | rs387906450 | n.l. | ID 10253 | pathogenic |
| sevHA15 | n.a. | Exon 15-16 deletion^ | Deletion/ Insertion | 1 patients reported with severe HA | n.l. | n.l. | n.l. | pathogenic |
| sevHA16 | IVS 15+1(G>A) | c.5373+1G>A | Splice | 5 patients reported with severe HA | n.l. | n.l. | n.l. | pathogenic |
| sevHA17 | Combined big deletion/insertionen in exon 1 | Combined big deletion/insertionen in exon 1 | Deletion/ Insertion |  |  |  |  | pathogenic |
| sevHA18 | Intron 22 inversion | Intron 22 inversion | Intron 22 |  |  |  |  | pathogenic |
| sevHA19 | Intron 22 inversion | Intron 22 inversion | Intron 22 |  |  |  |  | pathogenic |
| sevHA20 | Intron 22 inversion | Intron 22 inversion | Intron 22 |  |  |  |  | pathogenic |
| sevHA21 | IVS 15+1 (G>A) | c.5373+1G>A | Splice | 5 patients reported severe HA | rs1387946073 | n.l. | n.l. | pathogenic |
| sevHA22 | c.2188T>C, p.Cys711Arg | c.2188T>C, p.Cys730Arg | Missense | n.l. | n.l. | n.l. | n.l. | likely pathogenic |
| sevHA23 | Intron 22 inversion | Intron 22 inversion | Intron 22 |  |  |  |  | pathogenic |
| mildHA24 | c.2119T>C, p.Trp707Arg | c.2119T>C, p.Trp707Arg | Missense | 2 patients reported with mild HA | n.l. | n.l. | n.l. | pathogenic |
| modHA25 | c.1009G>T, p.Asp318Tyr | c.1009G>T, p.Asp337Tyr | Missense | 1 patient reported without severity | n.l. | n.l. | n.l. | likely pathogenic |
| modHA26 | c.5398C>T, p.Arg(CGT1781Cys(TGT) | c.5398C>T, p.Arg1800Cys | Missense | 76 patients reported, predominantly moderate | rs137852442 | n.l. | ID10274 | pathogenic |
| modHA27 | c.4380delT, p.Asn1460Lysfs | c.4380delT, p.Asn1460Lysfs*5 | Deletion/ Insertion | 7 cases with predominantly moderate HA | rs2073178986 | n.l. | n.l. | pathogenic |
| modHA28 | c.6518C>T, p.Thr2173Ile | c.6518C>T, p.Thr2173Ile | Missense | listed as moderate to severe | rs1603263397 | n.l. | ID 811508 | pathogenic |
| modHA29 | c.6518C>T, p.Thr2173Ile | c.6518C>T, p.Thr2173Ile | Missense | listed as moderate to severe | rs1603263397 | n.l. | ID 811508 | pathogenic |
| modHA30 | Arg(CGT)2150His(CAT) | c.6506G>A, p.Arg2169His | Missense | 182 patients reported, predominantly moderate | rs137852461 | n.l. | ID 10315 | pathogenic |

Table S4: Genetic revaluation for patients with HB

In original genetic reports protein position is often given as position in mature FVIII without signal peptide (minus 28 aa) revaluation using MANE Select Transcripts NM_000133.4(F9); ?, reclassification not possible; n.l., not listed; data base query 04/2025

| **ID** | **Original genetic information** | **Revaluation** | **Distribution of genetic alterations (Figure 3)** | **EAHAD** | **dbSNP v156** | **gnomAD v2.1.1** | **Clinvar** | **ACMG**  **classification** |
| --- | --- | --- | --- | --- | --- | --- | --- | --- |
| sevHB31 | Exon deletion f-h | ? | Deletion/ Insertion |  |  |  |  | pathogenic |
| sevHB32 | p.Trp310Ser | c.1067G>C,  p.Trp356Ser | Missense | listed with severe HB | n.l. | n.l. | n.l. | pathogenic |
| sevHB33 | c.88+1G>T | c.88+1G>T | Splice | listed as moderate to severe HB | rs1603263397 | n.l. | ID 811508 | pathogenic |
| sevHB34 | c.88+1G>T | c.88+1G>T | Splice | listed as moderate to severe | rs1603263397 | n.l. | ID 811508 | pathogenic |
| sevHB35 | c.88+1G>T | c.88+1G>T | Splice | listed as moderate to severe | rs1603263397 | n.l. | ID 811508 | pathogenic |
| modHB36 | c.1147C>G, p.Leu383Val | c.1147C>G, p.Leu383Val | Missense | not listed (amino acid position listed with Leu383Phe and Leu383Ile in a total of 7 patients with moderate to severe HB) | rs1677128088 VUS | n.l. | ID1061742 VUS | VUS |
| modHB37 | IVS 5+13A>G | c. 520+13A>G | Splice | 35 patients reported, predominantly moderate to mild | rs1603265507 | n.l. | ID627180 | pathogenic |
| modHB38 | IVS 5+13A>G | c. 520+13A>G | Splice | 35 patients reported, predominantly moderate to mild | rs1603265507 | n.l. | ID627180 | pathogenic |
| modHB39 | p.Arg248Gln | c.881G>A,  p.Arg294Gln | Missense | 140 patients reported, predominantly moderate | rs137852249 | n.l. | 10602 | pathogenic |

Table S5: Genetic revaluation for patients with vWD type 3

| **ID** | **Original genetic information** | **Revaluation** | **EAHAD** | **dbSNP v156** | **gnomAD v2.1.1** | **Clinvar** | **ACMG**  **classification** |
| --- | --- | --- | --- | --- | --- | --- | --- |
| vWE40 | c.3285_3307delinsTCC, p.Asp1096Profs*39 | c.3285_3307delinsTCC, p.Asp1096Profs*39 | - | rs2136418437 | n.l. | ID1065298 | pathogenic |
| vWE41 | c.3285_3307delinsTCC, p.Asp1096Profs*39 | c.3285_3307delinsTCC, p.Asp1096Profs*39 | - | rs2136418437 | n.l. | ID1065298 | pathogenic |

# Supplementary Figures

## Figure S1: Detailed information about the distribution of osteochondral and soft tissue subcategories in ankle


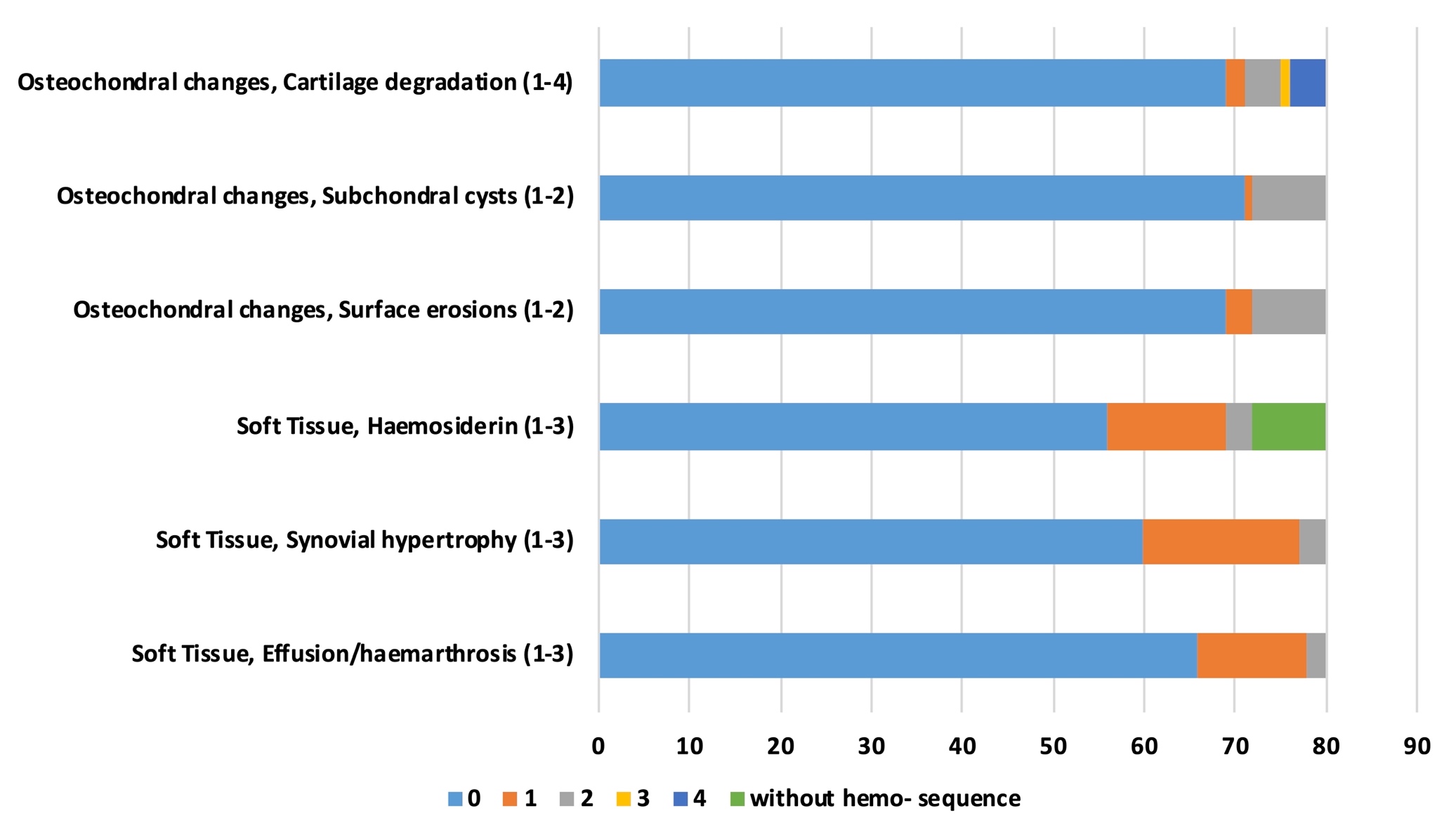


Figure S2: Detailed information about the distribution of osteochondral and soft tissue subcategories in knee


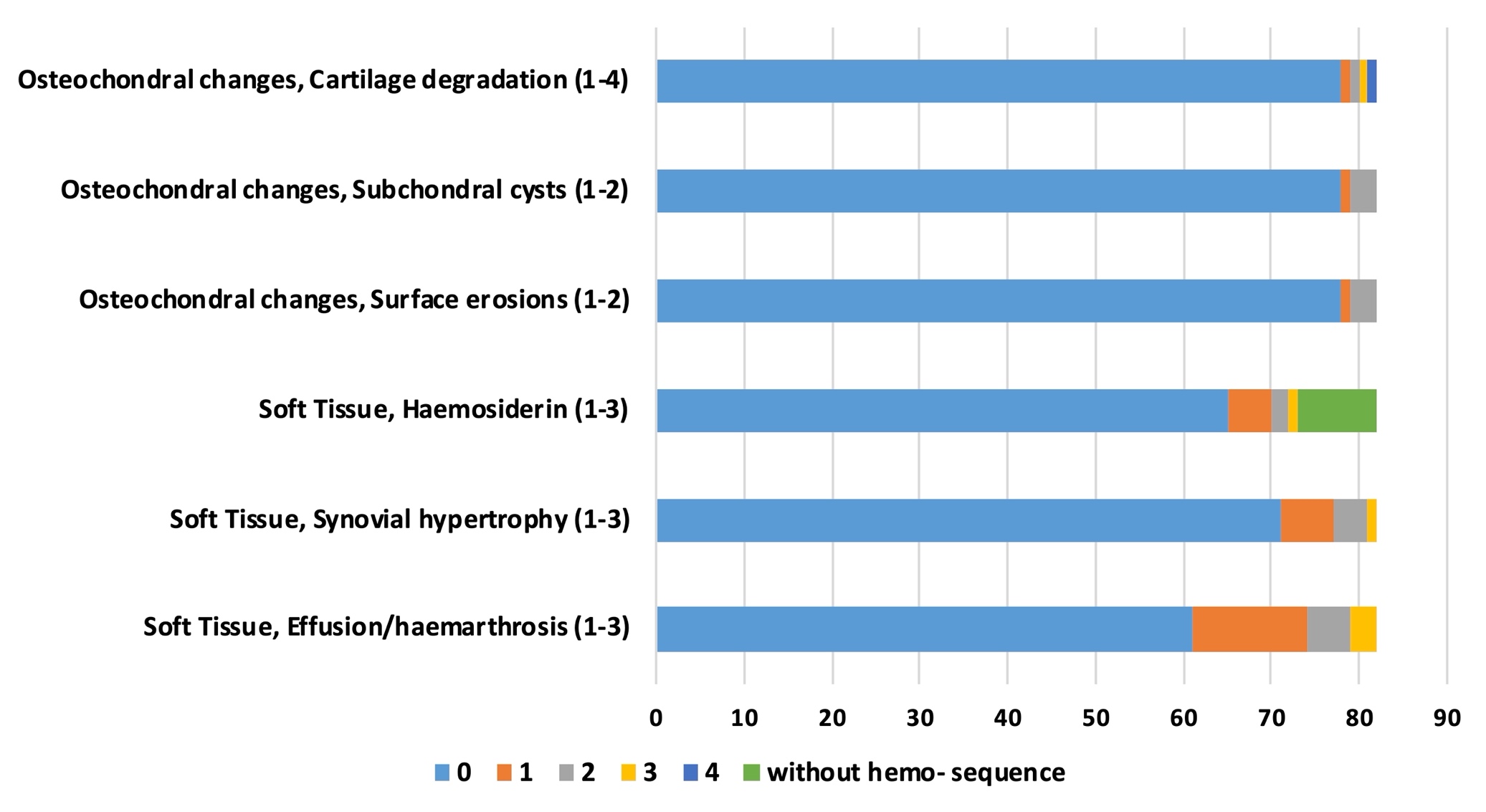


Figure S3: Detailed information about the distribution of osteochondral and soft tissue subcategories in elbow


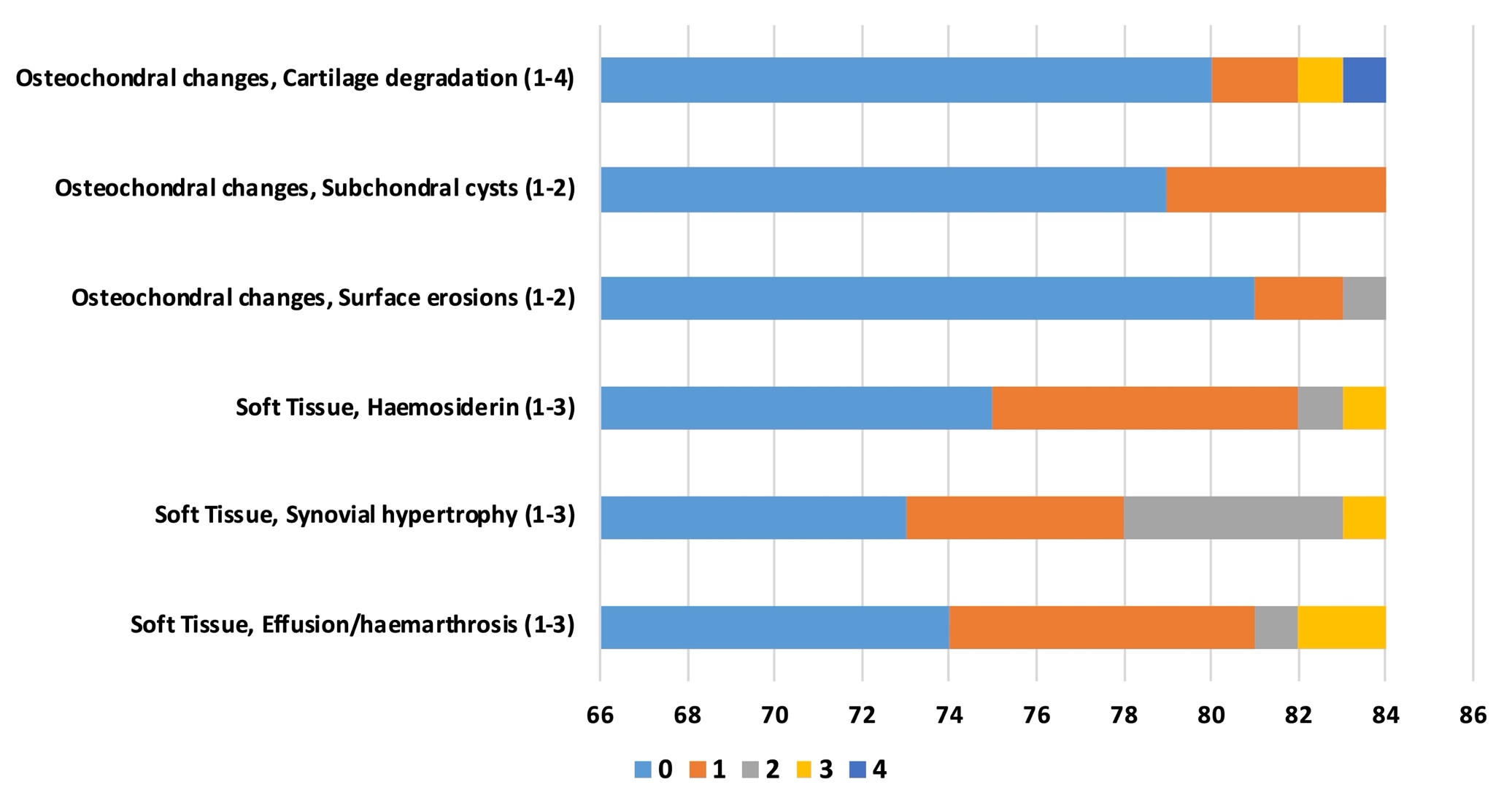

Supplement: Supplementary file 1 [file Supplementary_file_1.docx]
